# Diagnostic Accuracy of the Enhanced Liver Fibrosis (ELF^®^) Score Using HCV-Infected Serum Samples Cryopreserved for up to 25 Years

**DOI:** 10.1371/journal.pone.0164883

**Published:** 2016-12-16

**Authors:** Marc Puigvehí, Juanjo Hernández, Teresa Broquetas, Susanna Coll, Montserrat Garcia-Retortillo, Nuria Cañete, Maria Dolors Giménez, Mar Garcia, Felipe Bory, Margarita Salvadó, Ricard Solà, José A. Carrión

**Affiliations:** 1 Liver Section, Gastroenterology Department, Hospital del Mar, Universitat Autònoma de Barcelona; 2 IMIM (Hospital del Mar Medical Research Institute), Barcelona, Spain; 3 Laboratori de Referència de Catalunya, Barcelona, Spain; 4 Pathology Department, Hospital del Mar, Barcelona, Spain; Medizinische Fakultat der RWTH Aachen, GERMANY

## Abstract

**Introduction & Aims:**

Cryopreservation of serum samples is a standard procedure for biomedical research in tertiary centers. However, studies evaluating the long-term biological stability of direct liver fibrosis markers using cryopreserved samples are scarce.

**Methods:**

We compared the stability of hyaluronic acid (HA), tissue inhibitor of metalloproteinases (TIMP-1) and amino-terminal propeptide of type III procollagen (PIIINP) in 225 frozen serum samples of HCV-infected patients with a paired liver biopsy for up to 25 years (1990–2014). Moreover, we assessed the diagnostic accuracy (AUROC) of the Enhanced Liver Fibrosis (ELF^®^) score to identify significant fibrosis (F2-4) and its predictive capacity to identify clinical events during follow-up.

**Results:**

Seventy-six patients (39,8%) had mild fibrosis (F0-1) and 115 (60,2%) significant fibrosis (F2-4). HA, PIIINP and TIMP-1 values remained stable during the period from 1995 to 2014 while those of 1990–94 were slightly higher. We did not find significant differences in the median ELF^®^ values during the 20-year period from 1995–2014 in patients with mild (from 8,4 to 8,7) and significant fibrosis (from 9,9 to 10,9) (p = ns between periods and fibrosis stages). The AUROCs of ELF^®^ to identify significant fibrosis were high in all the periods (from 0,85 to 0,91). The ELF^®^ score showed a good predictive capability to identify clinical events during follow-up.

**Conclusions:**

The biological stability of direct serum markers (HA, PIIINP and TIMP-1) using HCV-infected samples cryopreserved for 20 years is good. Therefore, the diagnostic accuracy of the ELF^®^ score to identify significant fibrosis and clinical events during follow-up is very high.

## Introduction

Non-invasive methods involving serum markers or radiological techniques are an essential diagnostic tool to assess liver fibrosis during patient’s follow-up [1] In patients with chronic hepatitis C virus (HCV) infection, significant fibrosis (F2-4) requires the need to start antiviral therapy [2]. Although transient elastography has shown high applicability when performed by experienced operators using the most adequate probe [3], serum markers have the highest applicability. Indirect serum markers are widely used in clinical practice for monitoring patients during follow-up. Moreover, serum markers have demonstrated a good correlation with liver fibrosis stage [4], showing a high diagnostic accuracy to predict histological progression and clinical outcomes [5].

During the last 15 years many fibrosis markers showing good precision to identify or to exclude significant (F2-4) or advanced fibrosis (F3-4) or cirrhosis (F4) have been proposed. Direct serum markers are proteins involved directly in the synthesis or degradation of hepatic extracellular matrix [6]. Hyaluronic acid (HA) is a glycosaminoglycan produced by hepatic stellate cells involved in fibrogenesis; Amino-terminal propeptide of type-III-procollagen (PIIINP) is a marker of inflammation and early fibrogenesis, and tissue inhibitor of metalloproteinase type-1 (TIMP-1) inhibits matrix metalloproteinase, thereby worsening fibrogenesis. The Enhanced Liver Fibrosis (ELF^®^) score is a panel combining these three direct markers (HA, TIMP-1 and PIIINP) that have demonstrated a good correlation with liver fibrosis stage [7–9]. The serum samples used to calculate these markers are often cryopreserved for biomedical research in tertiary centers. However, biomarkers may potentially lose stability due to protein degradation during the storing procedure, which may lead to the observation of lower levels when analyzed after several years [10]. This is crucial in studies evaluating fibrosis progression. Moreover, the recommended storage temperature remains unclear.

Frozen serum samples have demonstrated good stability to identify different biochemical parameters when preserved at -30°C to -80°C [11, 12]. Indeed, storage at -25°C produces a large variation in the stability of different serum components, and is thus considered an unsuitable temperature [13]. Therefore, the stability of serum biomarkers cryopreserved in long-term storage remains controversial. The scarce studies that have evaluated the long-term stability of proteins used tests analyzing accelerated stability, such as the Arrenhius equation [10], to obtain results in a short period of time. Another variable to take into account before analyzing cryopreserved serums is the number of freezing/thawing cycles the samples undergo since freezing/thawing can modify the stability of high molecular weight proteins and polysaccharides.

Thus, the primary aim of our study was to determine the reliability of serum samples of HCV-infected patients cryopreserved over a long period of time to assess significant fibrosis using HA, PIIINP, TIMP-1 and the Enhanced Liver Fibrosis (ELF^®^) score. The secondary aim of our study was to confirm the diagnostic accuracy of the ELF^®^ score to identify clinical events during a follow-up of 25 years.

## Patients and Methods

### Serum samples & liver biopsies

Serum samples and paired liver biopsies of HCV-infected patients cryopreserved from 1990 to 2014 were considered for the study. All the serum samples had been extracted in fasting conditions and centrifuged at 3000 rpm before preservation at -30°C. Serum samples were part of our private collection (C.0000956) of the IMIM (Hospital del Mar Medical Research Institute). The serum samples were identified with a number, and all the data were collected and tabulated in a database with an access code to ensure patient confidentiality. The study protocol was approved by the Ethical Committee of our institution “Comitè Ètic d’Investigació Clínica (CEIC)- Parc de Salut Mar”, study reference 2015/6161/I, in accordance with the ethical guidelines of the 1975 Declaration of Helsinki. Patients enrolled from 1990 to May 2006 gave oral informed consent for the use of serum samples in biomedical research, and it was collected in clinical history and electronic medical records. Those enrolled from May 2006 to 2014 provided written informed consent.

The samples were selected according to the date of collection. We considered 5-year periods (1990–94, 1995–99, 2000–2004, 2005–2009 and 2010–2014). Fifty samples were randomly selected from each period with the exception of the first period from which only 25 samples were selected due to the low availability of liver biopsies. The serum samples excluded were: those which had undergone a thawed cycle, those collected later than 24 months after the date of liver biopsy, and those with a fragmented liver biopsy or with less than 6 portal tracts or 10 mm in length.

A single blinded expert pathologist (M.G) revised all the biopsies and scored liver fibrosis stage according to the Metavir classification [14].

### Biochemical analysis

Frozen serum samples were transported in frozen conditions using dry ice, and the thawing process was performed in the “Laboratori de Referència de Catalunya S.A, Barcelona”. The ELF^®^ score is a panel combining three direct markers (HA, TIMP-1 and PIIINP) [9]. These three markers were analysed by heterogeneous chemiluminescent sandwich-type immunoassays using paramagnetic particles and pairs of monoclonal antibodies with acridinium ester labels and FITC respectively, to capture and quantify PIIINP and TIMP1, or HA binding protein (HABP) for HA analysis. Automatic XP and CP analysers were used to analyse quality reagents, calibrators and control materials manufactured and marketed, by Siemens Healthcare Diagnostics and Siemens ADVIA CENTAUR. Total standardized variation coefficients (intra- and interserial) along the entire analytical range did not exceed 7,4% for HA, 6,5% for PIIINP, and 7,3% for TIMP1. The detection ranges were: 1,6–1000 ng/mL for HA, 0,5–150 ng/mL for PIIINP and 3,5–1300 ng/mL for TIMP1. ELF^®^ was automatically generated with an immunochemical analyser (XP and CP SIEMENS ADVIA CENTAUR) using the equation provided by the manufacturer (2,494 + Index 0,846 ln [HA ng/mL] + 0,735 ln [PIIINP ng/mL] + 0,391 ln [TIMP1 ng/mL]).

Bilirubin, alanine aminotransferase (ALT), hemoglobin (Hb), albumin, platelets and international normalized ratio (INR) values, as well as validated indices to assess liver fibrosis [the AST to platelet ratio index (APRI) [15], Forns index [16] and FIB-4 [17]] were calculated using data from fresh blood samples and that obtained from electronic medical records according to previously published formulas, and in all cases from blood analysis within ± 3 months from the date of biopsy.

### Study design and end points

The stability of the cryopreserved samples was assessed by comparing the fibrosis markers in different periods and in patients with mild (F0-1) and significant fibrosis (F2-4). The interpretation of the liver fibrosis stage was performed based on previously published values according to the manufacturer's recommendations: ELF <7,7: no fibrosis (or mild); ELF ≥7,7 to <9,8: moderate fibrosis; ELF ≥9,8: severe fibrosis. A new cutoff of ELF ≥11,3 has recently been described to discriminate cirrhosis [8], and was also analysed. Diagnostic accuracy was defined as the capacity to identify significant fibrosis (METAVIR F2-4).

Follow-up of the patients included was performed using data from electronic medical records. Cirrhosis during follow-up was diagnosed with the appearance of one of the following: thrombocytopenia < 130.000 and splenomegaly during at least 6 months with no other etiology; altered liver structures on abdominal ultrasound; the presence of oesophageal varices or portal hypertensive gastropathy on upper endoscopy; or METAVIR F4 on histological evaluation. Clinical events during follow-up were defined as clinical decompensation (ascites, hepatic encephalopathy, spontaneous bacterial peritonitis or portal hypertension-related bleeding), hepatocellular carcinoma or liver-related death.

### Statistical analysis

The sample size to evaluate differences between the diagnostic accuracy of indirect serum markers was estimated on statistical assumptions based on previous data [4, 15, 16, 17]; 186 patients were necessary to achieve significant differences between the AUROCs with an alpha risk of 0,05, a beta risk of 0,10 and a 10% missing rate of data, assuming a correlation with the positive and negative groups of 0,70. Quantitative variables are expressed as medians (range). Differences between qualitative variables were assessed with the Fisher exact test and quantitative variables were analyzed with a non-parametric test (Mann-Whitney or Kruskal-Wallis for independent samples) in the different study periods and stages of fibrosis. The diagnostic accuracy of ELF^®^, Forns Index, APRI and FIB-4 to identify significant fibrosis in different periods was assessed with the Area Under the Receiver Operator Curve (AUROC) and their previously validated cutoffs according to sensitivity (S), specificity (Sp), positive predictive value (PPV), negative predictive value (NPV) and likelihood ratio (LR). *Comparison between ELF*^*®*^ *and indirect fibrosis markers (Forns*, *APRI and FIB-4) was performed to show non-inferiority of ELF*^*®*^. The predictive capacity of ELF^®^ for previously published cutoffs [8] to identify clinical events during follow-up was calculated using Kaplan-Meier curves. Differences in the baseline characteristics between patients with or without clinical events during follow-up were evaluated by univariate analysis. Variables showing a p value < 0.05 were included in a multivariate forward stepwise logistic regression analysis to identify the independent predictors of clinical events during follow-up. Comparisons between AUROCs were made using the method of Hanley and McNeil [18] and calculated with MedCalc^®^ v12.5.0 (MedCalc Software, Mariakerke, Belgium). The remaining statistical analyses were performed with SPSS® 19.0 (SPSS Inc., Chicago IL). All data necessary to reproduce the results of this study have been provided within the manuscript and supporting information files (S1 Database).

## Results

### Baseline characteristics of the patients included

Two hundred and twenty-five samples from HCV-infected patients were considered for the study (50 samples from five-year periods between 1995 and 2014, and 25 samples from the 1990–94 period). Seven (3,1%) samples were excluded because of the low-quality of liver biopsy and 27 (12%) for having more than 2 years between serum collection and liver biopsy. Thus, 191 samples were finally included. The baseline characteristics of the patients are summarized in Table 1.

**Table 1:** Characteristics according to fibrosis stage in HCV-infected patients (N = 191).

	METAVIR 0–1 (Mild fibrosis) (n = 76, 39,8%)	METAVIR 2–4 (Significant fibrosis) (n = 115, 60,2%)	p
**Age (years)**	**40,5 (18,1–67,2)**	**47,7 (23,5–73,1)**	**<0,001**
**Gender (male, %)**	47 (61,8)	80 (69,6)	ns
**Bilirubin (mg/dL)**	**0,7 (0,1–2)**	**0,8 (0,3–2,2)**	**0,024**
**ALT (IU)**	**56 (15–845)**	**115 (13–553)**	**<0,001**
**Albumin (g/dL)**	**4,4 (3,5–5,1)**	**4,3 (3–5)**	**0,002**
**Platelets (10**^**9**^**)**	**222 (71–373)**	**165 (48–385)**	**<0,001**
**INR**	**1,01 (0,82–1,25)**	**1,04 (0,9–1,65)**	**<0,001**
Hb **(g/dl)**	14,8 (10,7–18)	14,9 (10,2–17,7)	ns
**ELF® score**	**8,5 (7,2–12,1)**	**10,3 (8–14,1)**	**<0,001**
**Forns index**	**3,8 (-0,2–9)**	**6,5 (2,1–11,4)**	**<0,001**
**APRI**	**0,5 (0,2–12)**	**1,3 (0,2–6,6)**	**<0,001**
**FIB-4**	**0,97 (0,3–13,1)**	**2,4 (0,6–16,8)**	**<0,001**
**Length of LB (mm)**	**20 (13–40)**	**16 (10–34)**	**<0,001**

HCV, hepatitis C virus; ALT, alanine aminotransferase; INR, International Normalized Ratio; Hb, hemoglobin; ELF, Enhanced Liver Fibrosis; APRI, AST to **p**latelet **r**atio index; LB, liver biopsy

Patients were divided according to the presence or absence of significant fibrosis, considering the importance of this classification in clinical practice. Seventy-six patients (n = 76, 39,8%) had mild fibrosis (METAVIR F0-1) and 115 (60,2%) significant fibrosis (METAVIR F2-4). Patients with significant fibrosis were older, had lower albumin levels and platelet counts, and higher bilirubin, ALT, and INR values and fibrosis markers compared to those with mild fibrosis (p < 0,05 all). We did not find significant differences in the proportion of patients for each fibrosis stage (F0-4) in the different periods (Table 2).

**Table 2:** Distribution of HCV-infected patients in every single fibrosis stage (N = 191).

PERIOD N (%) METAVIR N (%)	1990–94 (n = 18, 9,4%)	1995–99 (n = 37, 19,4%)	2000–04 (n = 44, 23%)	2005–09 (n = 45, 23,6%)	2010–14 (n = 47, 24,6%)	p
**F0 (n = 36, 18,8%)**	1 (5,6)	8 (21,6)	9 (20,5)	8 (17,8)	10 (21,3)	ns
**F1 (n = 40, 20,9%)**	4 (22,2)	8 (21,6)	8 (18,2)	10 (22,2)	10 (21,3)	ns
**F2 (n = 43, 22,5%)**	4 (22,2)	10 (27)	8 (18,2)	10 (22,2)	11 (23,4)	ns
**F3 (n = 34, 17,8%)**	4 (22,2)	5 (13,5)	10 (22,7)	10 (22,2)	5 (10,6)	ns
**F4 (n = 38, 19,9%)**	5 (27,8)	6 (16,2)	9 (20,5)	7 (15,6)	11 (23,4)	ns

### Bio-stability of direct fibrosis markers and the ELF^®^ score during long periods

The median values of the serum biomarkers (HA, PIIINP, TIMP-1) and the ELF^®^ score according to the fibrosis stage in each 5-year time period are depicted in Table 3. Among patients with mild fibrosis (n = 76, 39,8%) the median HA (from 20,2 to 24,4), PIIINP (from 6,1 to 7,5) and TIMP-1 values (from 182,1 to 215,1) were stable in the periods from 1995 to 2014 (all p = ns). Moreover, the median values of the ELF^®^ score were nearly equal (from 8,4 to 8,7) in 1995–2014 periods (p = ns). The median HA and TIMP-1 values in the first period studied (1990–94) were similar, with only PIIINP (13,9) differing (p = 0,009). However, the ELF^®^ score remained stable in the first period (9,6; p = ns).

**Table 3:** Serum markers (HA, PIIINP, TIMP-1) and ELF® values according to fibrosis stage in each 5-year time period.

	1990–94 (n = 18, 9,4%)	1995–99 (n = 37, 19,4%)	2000–04 (n = 44, 23%)	2005–09 (n = 45, 23,6%)	2010–14 (n = 47, 24,6%)	p^1^	p^2^
**Mild Fibrosis (F0-1) (n = 76, 39,8%)**	**N = 5**	**N = 16**	**N = 17**	**N = 18**	**N = 20**		
**HA**	**43,1** (31,7–128,2)	**24,4** (8–535,5)	**21,5** (8,3–300,1)	**20,2** (7,6–258,6)	**23,6** (10,5–106,3)	ns	ns
**PIIINP**	**13,9** (9,9–20,2)	**7,5** (4,1–15,2)	**6,1** (3,7–21,8)	**6,5** (4,2–10,3)	**6,6** (4,1–11,8)	**0,009**	ns
**TIMP-1**	**190,6** (159,3–296,8)	**212,4** (83,8–339,3)	**215,1** (96,6–389,9)	**184,7** (64,6–277,1)	**182,1** (63,5–349,9)	ns	ns
**ELF**	**9,6** (9,2–11)	**8,7** (7,2–12,1)	**8,5** (7,4–11,9)	**8,4** (7,2–10,5)	**8,5** (7,5–10,2)	ns	ns
**Significant Fibrosis (F2-4) (n = 115, 60,2%)**	**N = 13**	**N = 21**	**N = 27**	**N = 27**	**N = 27**		
**HA**	**356,8** (31,7–2179,6)	**83,2** (26,3–895,4)	**84,2** (14,8–979,7)	**73,5** (15,3–1066,6)	**135,6** (26,5–780)	**0,047**	ns
**PIIINP**	**13,3** (10–44,2)	**10,2** (4,2–29,5)	**11,9** (4,6–25,7)	**9,9** (5,7–32,7)	**11,2** (4,7–30,1)	**0,041**	ns
**TIMP-1**	**370** (213,7–869,3)	**285,9**(109,2–408,9)	**356,8** (198,6–550,1)	**290,4** (127,9–624,9)	**353,2** (123,6–484,9)	**0,054**	ns
**ELF**	**11,7** (9,2–14,1)	**10,1** (8,6–13)	**10,3** (8–13,1)	**9,9** (8,5–12,8)	**10,9** (8,6–12,6)	**0,027**	ns

Among patients with significant fibrosis (n = 115, 60,2%) the median HA (from 73,5 to 135,6), PIIINP (from 9,9 to 11,9), and TIMP-1 values (from 285,9 to 356,8) and ELF^®^ (from 9,9 to 10,9) were stable from 1995 to 2014 (p = ns all). Samples from the first period (1990–94) showed higher median HA (356,8; p = 0,047), PIIINP (13,3; p = 0,041) and TIMP-1 values (370; p = 0,054), and ELF^®^ (11,7; p = 0,027).

### Diagnostic accuracy of ELF^®^ to identify significant fibrosis (F2-4) using cryopreserved serum samples

**Fig 1:**
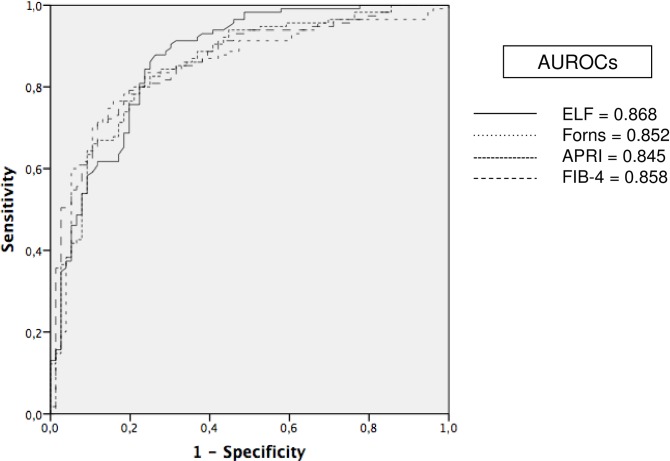
Diagnostic accuracy (AUROCs) of indirect (Forns, APRI, FIB-4) and direct (ELF) scores to identify significant fibrosis (F2-4).

We calculated the AUROC of the ELF^®^ score to identify significant fibrosis (METAVIR F2-4) in cryopreserved serums. Including all the study periods (n = 191) the AUROC to identify significant fibrosis was 0,868 (Fig 1). No significant differences were observed between the AUROCs (95% CI) of indirect serum markers in any period of time (Table 4). Moreover, on comparing the AUROCs of the ELF^®^ score among the different periods no significant differences were found ranging from 0,853 to 0,911 (p = ns). The AUROCs of the Forns Index, APRI and FIB-4 including all the periods were 0,852, 0,845 and 0,858, respectively (Fig 1). On the other hand, the AUROCs of the Forns Index for the different periods ranged from 0,786 to 0,883, being from 0,769 to 0,887 for the APRI; and from 0,805 to 0,906 for FIB-4.

**Table 4:** Diagnostic accuracy (AUROCs, 95% CI) of indirect (Forns, APRI, FIB-4) and direct (ELF) scores (N = 191) to identify significant fibrosis in each 5-year time period.

AUROCs n (%)	ELF^®^	FORNS	FIB-4	APRI	p
**1990–94 18 (9,4)**	0,854 (0,676–1)	0,815 (0,574–1)	0,831 (0,529–1)	0,769 (0,424–1)	ns
**1995–99 37 (19,4)**	0,869 (0,739–0,999)	0,786 (0,636–0,935)	0,863 (0,744–0,982)	0,887 (0,773–1)	ns
**2000–04 44 (23)**	0,866 (0,740–0,992)	0,882 (0,785–0,980)	0,906 (0,814–0,998)	0,869 (0,760–0,979)	ns
**2005–09 45 (23,6)**	0,853 (0,739–0,967)	0,842 (0,722–0,962)	0,805 (0,672–0,940)	0,800 (0,660–0,940)	ns
**2010–14 47 (24,6)**	0,911 (0,831–0,992)	0,883 (0,781–0,986)	0,822 (0,705–0,939)	0,819 (0,696–0,941)	ns

Using the previously validated cutoffs of ELF^®^ score, we found a good diagnostic accuracy of frozen serum samples to identify significant fibrosis with a positive predictive value (PPV) of 84% and specificity (Sp) of 80,3% similar to FIB-4 calculated with fresh blood samples (87% and 82,9%, respectively) (p = ns in both cases) (Table 5). In contrast, Forns Index showed good negative predictive value (NPV) and sensitivity (Se) (78,3% and 88,7%) similar to APRI (75,5% and 88,7%, respectively) (p = ns in both cases).

**Table 5:** Diagnostic accuracy (PPV, NPV, Se, Sp) of indirect (Forns, APRI, FIB-4) and direct (ELF) scores (N = 191) to identify significant fibrosis according to their previously validated cutoffs.

Validated cutoffs to identify F2-4 n (%)	F0-1 76 (39,8)	F2-4 115 (60,2)	PPV	NPV	Se	Sp	LR+
**ELF ≥ 9,8** ^**[**^^**8**^^**]**^**94 (49,2)**	15 (16)	79 (84)	84	62,9	68,7	80,3	3,5
**Forns ≥ 4,2**^**[**^^**16**^^**]**^**131 (68,6)**	29 (22,1)	102 (77,9)	77,9	78,3	88,7	61,8	2,3
**FIB-4 ≥ 1,45**^**[**^^**17**^^**]**^**100 (52,4)**	13 (13)	87 (87)	87	69,2	75,6	82,9	4,4
**APRI ≥ 0,5**^**[**^^**15**^^**]**^**138 (72,3)**	36 (26,1)	102 (73,9)	73,9	75,5	88,7	52,6	1,9

PPV, Positive Predictive Value; NPV, Negative Predictive Value; Se, Sensitivity; Sp, Specificity; LR+, Positive Likelihood Ratio

### Predictive capacity of the ELF^®^ score to identify clinical events during follow-up

**Fig 2:**
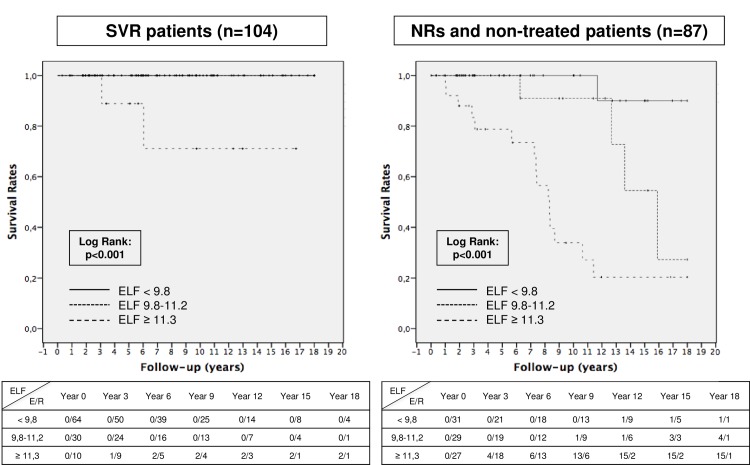
Predictive capacity of the ELF® score to identify clinical events during follow-up usingcryopreserved samples according to HCV-antiviral treatment response (n = 191). Data of number of events (E) and patients at risk (R) in every 3 years time period.

One hundred and seventy patients (n = 170, 89%) received HCV-antiviral treatment during follow-up. Among the treated patients, 104 (61,2%) achieved sustained virological response (SVR) and 66 (38,8%) were non-responders (NRs). On multivariate analysis, only SVR [OR = 0,06 (0,01–0,33), p = 0,002] was related to the absence of clinical events. Only 2 patients with SVR presented a clinical event during follow-up, with both patients developing hepatocellular carcinoma and having a high ELF^®^ score ≥ 11,3 (Fig 2).

As SVR was the only variable related with the development of clinical events, we divided the cohort into patients with or without SVR. Among NRs and non-treated patients (n = 87), 22 (25,3%) developed at least one clinical event during a median follow-up of 6,2 years. On multivariate analysis, the ELF^®^ score [OR = 2,31 (1,39–3,85), p = 0.001] and FIB-4 [OR = 2,09 (1,46–2,98), p = 0,001] were the only variables independently related to the probability of developing a clinical event during follow-up. According to the previously published cutoffs (8), only 3,2% (n = 1/31) patients with a low ELF^®^ score < 9.7 (n = 31, 35,6%) presented clinical events while 17,2% (n = 5/29) of the patients with intermediate ELF^®^ scores 9,8–11,2 (n = 29, 33,3%) presented events, and nearly 3 out of 4 cases (73,7%, n = 16/27) with a high ELF^®^ score ≥11,3 (n = 27, 31%) developed clinical events. Moreover, the event-free survival rate at 10 and 20-years of follow-up was 100% and 95% in patients with ELF^®^ scores < 9,7, 95% and 30% in patients with intermediate ELF^®^ (9,8–11,2), and 30% and 20% in those with high ELF^®^ scores ≥ 11,3 (Log Rank: p<0,001) (Fig 2).

## Discussion

To our knowledge this is the first study to describe the stability of direct fibrosis markers (HA, PIIINP and TIMP-1) in samples cryopreserved for more than 20 years. Moreover, we found that the diagnostic accuracy and predictive capacity of these cryopreserved samples remained unaltered following long-term storage. This is especially important for reference centers assessing fibrosis progression using retrospective samples.

In the present study we selected frozen samples from our biobank according to the date of cryopreservation rather than using an accelerated stability test [10] in order to evaluate the bio-stability of serum markers stored for up to 25 years. The median values of HA, PIIINP and TIMP-1 in patients with mild fibrosis remained highly stable over a period of 20 years. Moreover, HA, PIIINP and TIMP-1 values were higher in patients with significant fibrosis according to fibrosis stage. However, we observed increased HA and PIIINP values in the first period (1990–1994), while TIMP-1 values and the ELF^®^ score remained stable. Since a lack of stability during cryopreservation leads to sample degradation and decreased values [10], the differences in the first period are probably due to the low number of samples included. A larger number of samples is therefore necessary to make solid conclusions regarding stability after 20 years of cryopreservation. Importantly, the diagnostic accuracy of the ELF^®^ score was high (AUROC = 0,868), without differences in identifying significant fibrosis among the different periods, with AUROCs ranging from 0,853 to 0,911. Moreover, we did not find significant differences when compared ELF^®^ with indirect serum markers (Forns, FIB-4 and APRI) obtained from electronical medical records and calculated from fresh blood samples. Thus, a high diagnostic accuracy was observed, thereby confirming the stability of the biomarkers analysed (HA, PIIINP and TIMP-1) in frozen serum samples stored for up to 25 years.

Our secondary aim was to assess the predictive capacity of the ELF^®^ score using cryopreserved samples to identify patients at risk of developing clinical events during follow-up. The ELF^®^ score and FIB-4 were the only independent variables related to the probability of developing clinical events in NRs and non-treated patients. In our study, patients with a low ELF^®^ score < 9,7 showed no risk of developing clinical decompensation and the event-free rate was nearly 100% at 10 and 20 years of follow-up. However, intermediate values of the ELF^®^ score (9,8–11,2) showed high event-free rates during the first 10–15 years, but an acceleration of liver disease progression after this period. On the other hand, the low number of patients at risk, especially after 6–9 years of follow-up, limits solid decision making. These findings should be confirmed in prospective studies including a larger number of patients. Importantly, patients with a high ELF^®^ score ≥11,3 presented a high proportion of clinical events even during the first 5 years of follow-up. These results confirm the need for antiviral treatment in patients with ELF^®^ values > 9,7 and urgent treatment and close monitoring in those with an ELF^®^ score ≥ 11,3. Moreover, 2 out of 10 (20%) patients with SVR and an ELF^®^ score ≥ 11,3 developed hepatocellular carcinoma, thereby demonstrating the need for follow-up despite achieving SVR.

In conclusion, our study shows that analysis of high molecular weight substances such as direct fibrosis markers in frozen serum samples remain stable for at least 20 years. Moreover, the ELF^®^ score showed very good diagnostic accuracy to identify significant fibrosis in cryopreserved samples, being similar to or even better than the other indirect markers [15, 16, 17] analyzed in fresh samples. Finally, the predictive capacity of the ELF^®^ score to identify patients at risk of progression of liver disease is very high, being useful to recommend treatment initiation or close monitoring in HCV-infected patients.

## Supporting Information

S1 Database Database. Minimal necessary data to reproduce the study results.
(XLSX)

## Abbreviations

HCV: hepatitis C virus
HA: hyaluronic acid
PIIINP: amino-terminal propeptide of type III procollagen
TIMP-1: tissue inhibitor of metalloproteinases
ELF: enhanced liver fibrosis
HABP: hyaluronic acid binding protein
ALT: alanine aminotransferase
Hb: hemoglobin
INR: International Normalized Ratio
APRI: AST to **p**latelet **r**atio index
AUROC: Area Under the Receiver Operator Curve
PPV: Positive Predictive Value
NPV: Negative Predictive Value
Se: Sensitivity
Sp: Specificity
LR: Likelihood Ratio
SVR: sustained virological response
NRs: non-responders
